# Aseptic pyomyositis in rheumatoid arthritis treated with corticosteroid and DMARDs

**DOI:** 10.1093/omcr/omae059

**Published:** 2024-06-07

**Authors:** Sudhir Karmacharya, Adheep Arun Shrestha, Shweta Nakarmi, Manisha Bhochhibhoya, Binit Vaidya

**Affiliations:** Rheumatology Department, National Center for Rheumatic Diseases, House no 158, Pashupati Sadak, Kathmandu-9, 44600, Nepal; Rheumatology Department, National Center for Rheumatic Diseases, House no 158, Pashupati Sadak, Kathmandu-9, 44600, Nepal; Rheumatology Department, National Center for Rheumatic Diseases, House no 158, Pashupati Sadak, Kathmandu-9, 44600, Nepal; Rheumatology Department, National Center for Rheumatic Diseases, House no 158, Pashupati Sadak, Kathmandu-9, 44600, Nepal; Rheumatology Department, National Center for Rheumatic Diseases, House no 158, Pashupati Sadak, Kathmandu-9, 44600, Nepal

**Keywords:** pyomyositis, rheumatoid arthritis, immunosupression

## Abstract

Pyomyositis is a purulent infection of skeletal muscle that is mostly observed in tropical countries. Aseptic pyomyositis is a rare, potentially life-threatening disorder characterized by the formation of sterile pus in muscle. We present a case of 53-years old female, diagnosed case of seropositive rheumatoid arthritis, presented with pain and swelling of the right calf muscle for 2 weeks. There was no history of fever, cough, skin erythema, no history of prolonged standing or immobility, or fetal loss. The diagnosis was made as rheumatoid arthritis with autoimmune pyomyositis, and the patient was treated with oral prednisolone 1mg/kg body weight in tapering dose, cs DMARDS, (methotrexate 25 mg once a week, and leflunomide 20mg daily hydroxychloroquine 200 mg daily orally) and another supportive treatment along with surgical drainage of pus was done. There was complete resolution of the initial lesion and remission of the primary disease in 3 months.

## Introduction

Aseptic pyomyositis is a rare and potentially life-threatening disorder characterized by recurrent manifestation of deep necrotizing areas containing mature neutrophils that do not respond to antibiotics. It is suggested to be a part of the spectrum of auto-inflammatory disorders.

The main characteristics of aseptic pyomyositis include absence of microbes, bacteria, viruses, and parasites; and poor response to antibiotic therapy [1]. Rapid clinical improvement after corticosteroid therapy (with colchicine and DMARDs- Disease Modifying Anti-Rheumatic Drugs) is an important feature [1, 2]. It is usually associated with autoimmune inflammatory diseases like rheumatoid arthritis (RA), lupus, inflammatory bowel disease [2]. We describe a case of aseptic pyomyositis of the right calf muscle in a patient with RA, where the patient fully recovered after a high dose of glucocorticoid and DMARDs.

## Case presentation

**Figure 1:**
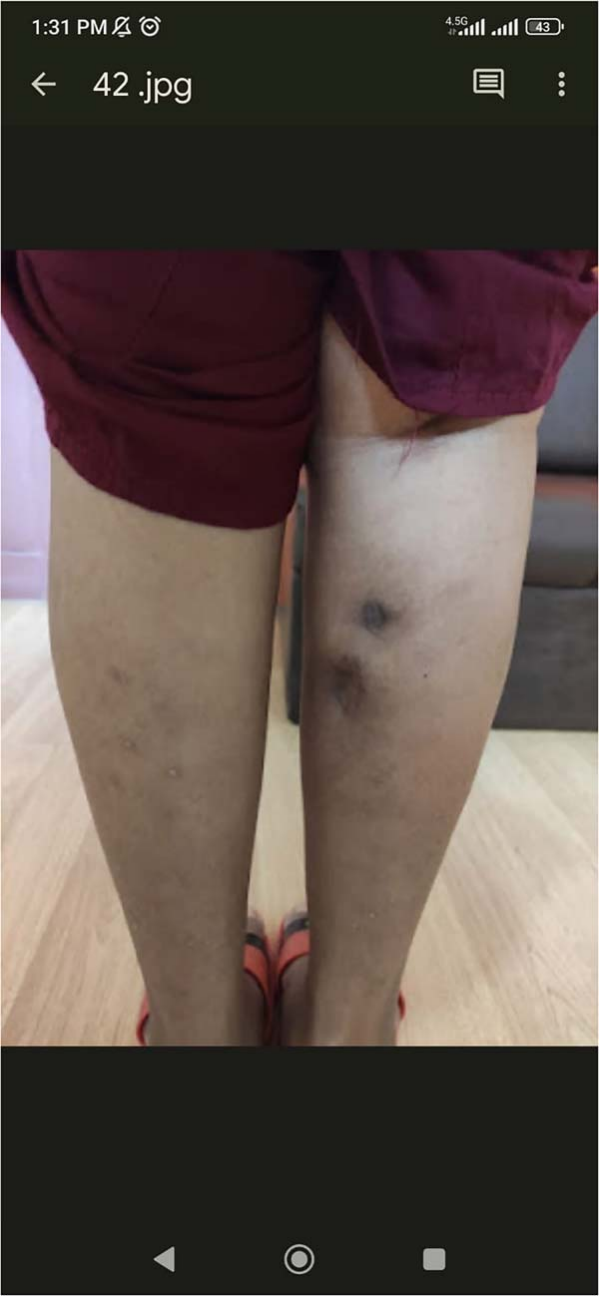
Scar mark present in right calf muscle indicating previous incision and drainage.

A 53-years old female, diagnosed case of rheumatoid arthritis, presented with pain and swelling of the right calf muscle for 2 weeks. There was no history of fever, cough, skin erythema, and prolong immobility. She was diagnosed as seropositive RA 9 years ago on the basis of inflammatory polyarthritis, raised acute phase reactants, positive anti-CCP antibodies. She was being treated with methotrexate and leflunomide with relapsing and remitting course of disease. Two episodes of similar presentation of pain and swelling of the right calf without fever occurred 2 years back at 6 months interval, for which incision and drainage was done and 1 liter of pus was drained. She was treated with parenteral antibiotics with poor recovery on both occasions. Physical examination revealed swelling and tenderness over her right calf (Fig. 1).

The overlying skin was warm but not erythematous. Her investigation reports are mentioned in Table 1. Diagnosis of autoimmune sterile pyomyositis was made, and the patient was treated with oral prednisolone 1 mg/kg/day on tapering dose and conventional DMARDS, (methotrexate 25 mg once a week, leflunomide 20 mg daily and hydroxychloroquine 200 mg daily). Pus drainage was not done at this setting. The patient was reviewed after 1 week with repeat sonography to assess the need for drainage. There was symptomatic improvement along with reduction in size of collection and the ongoing treatment was continued. There was complete resolution of the initial lesion and remission of the primary disease (RA) at 3 months Fig. 3.

**Figure 2:**
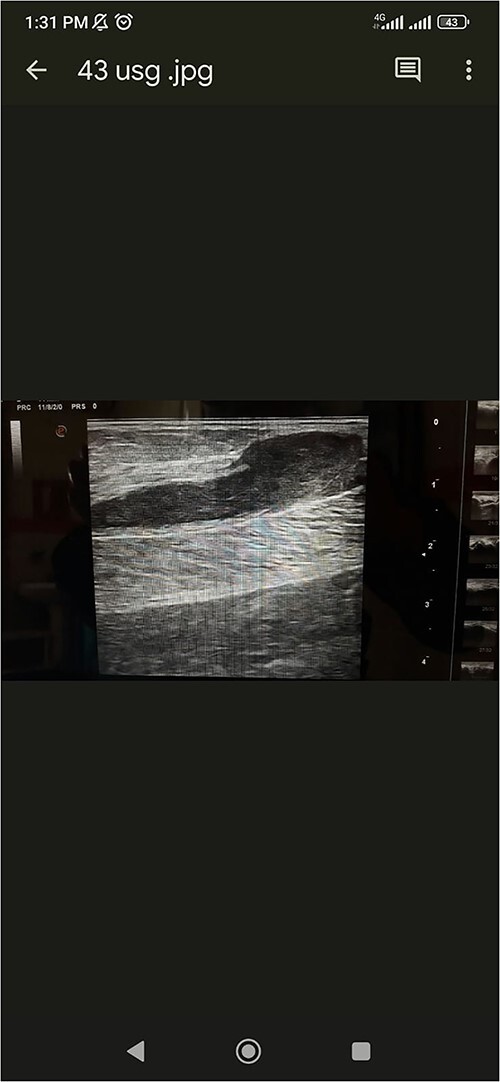
USG of right calf showing hypoechoic area in right medial gastrocnemius muscle.

**Figure 3:**
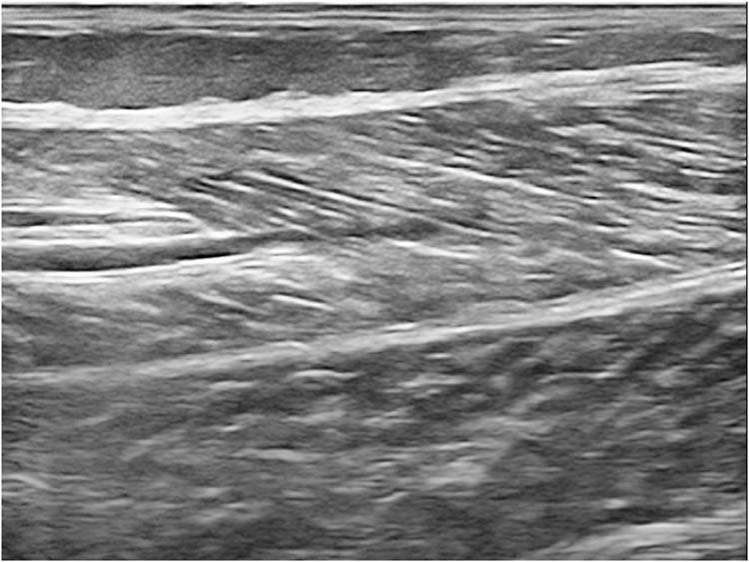
USG of right calf muscle after treatment with immunosuppressive.

**Table 1:** Investigations reports

Investigations	First episode 11/11/2021	Second episode 06/05/2022	At a time of presentation
Total count (/cumm)	5400	9800	8200
Neutrophil %	72	78	75
Hb (gm/dl)	12.1	11.6	11.2
Platelet (/cumm)	425 000	378 000	445 000
Blood C/S	Sterile	Sterile	Sterile
Urine R/M/E	Normal	Normal	Normal
Chest x-ray	NA	NA	Normal
SGPT (IU)	43	37	37
SGOT (IU)	32	36	42
S.albumin (g/dl)	NA	NA	4.2
S. creatinine (mg/dl)	0.6	0.8	0.9
S. procalcitonin (ng/dl)	NA	NA	0.03
S. lactate (mmol/L)	NA	NA	0.8
CRP (mg/L)	NA	48.2	33.42
ESR (mm 1st hr)	90	78	114
Gram staining	Negative	Negative	Negative
Ziehl-Neelsen staining	Negative	NA	Negative
Pus C/S (aerobic, anaerobic, fungal and mycobacterial)	Sterile	Sterile	Sterile
USG of right calf	NA	Inflammatory arthritis Localized collection in right gastrocnemius. Figure 2	Localized collection in right gastrocnemius with no doppler uptake. Negative for thrombosis.
MRI scan of right leg	NA	NA	Loculated collection in the medial head of gastrocnemius (4x4x1.5 cm; volume 12 cc), mild to moderate knee joint effusion, synovial thickening, patchy marrow edema in the lateral femoral condyle. Figs 4 and 5

NA: Not applicable, C/S: Culture/Sensitivity, R/M/E: Routein/Microscopy/Examination

**Figure 4:**
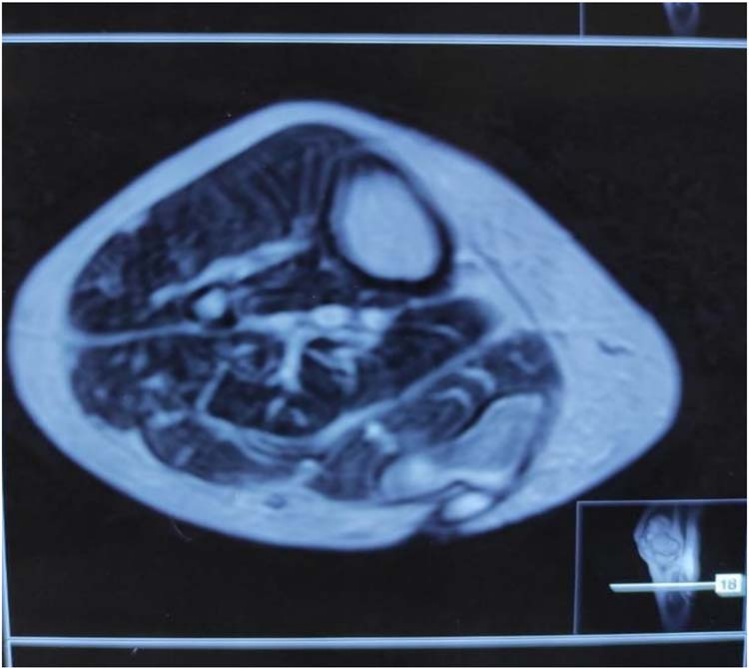
MRI of right calf muscle T2W image showing loculated collection over medial gastrocnemius muscle

**Figure 5:**
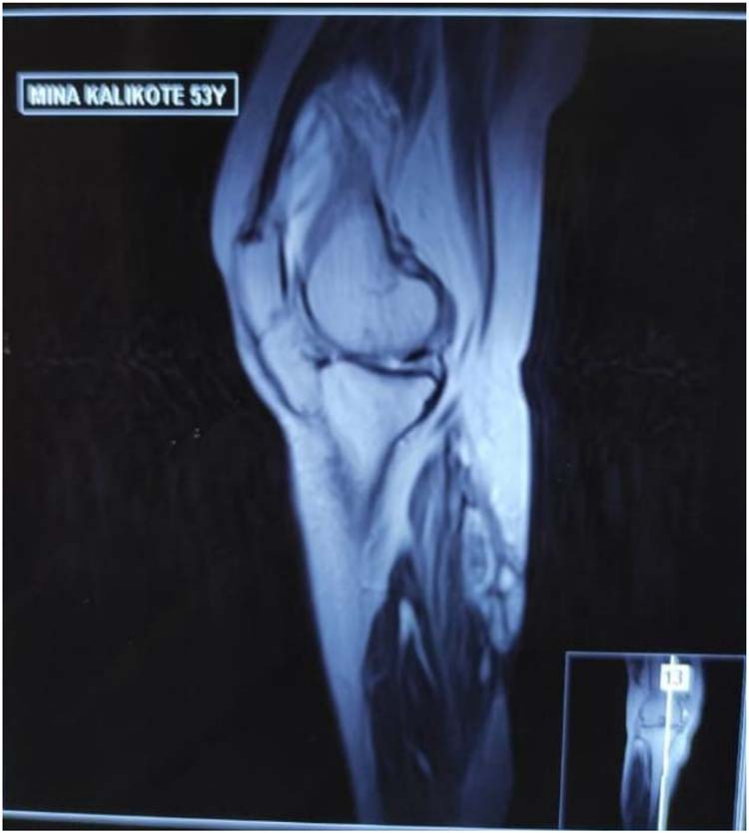
MRI of right calf muscle T2W image showing loculated collection over medial gastrocnemius muscle.

## Discussion

Sterile pyomyositis is a rare entity which is usually associated with autoimmune conditions like RA, lupus, Crohn’s disease, and ulcerative colitis [2]. Patients typically present with high disease activity or flare of the primary autoimmune condition with or without signs of infection and constitutional symptoms like weight loss, lethargy, fatigue [2].

The diagnosis of aseptic pyomyositis is sometimes delayed due to its vague presentation. This patient had rheumatoid arthritis and there was possibility of popliteal cyst (Baker’s cyst) rupturing in the calf muscle producing pain, swelling, and tenderness in the calf. This possibility was ruled out on recurrent involvement in calf muscles and on imaging. In addition, she improved after administration of high-dose glucocorticoid and DMARDs, supporting a high disease activity rather than infective etiology. A similar case of aseptic abscess syndrome has been described by Agirgol et al, in which a middle-aged man developed multiple abscesses in his internal organs along with constitutional symptoms like fever and weight loss. He was also treated successfully with steroid and colchicine [3]. A case series by Trefond et al., has also described 71 cases with aseptic abscesses in various sites mostly associated with autoimmune rheumatic diseases like rheumatoid arthritis, inflammatory bowel disease, pyoderma gangrenosum, spondyloarthropathies. Successful treatment was done with colchicine and DMARDs. However, 44 out of 71 had at least one relapse [2].

Diagnosis should always be done after exclusion of all other possibilities. In our case, infective pyomyositis was a strong differential as she was on DMARDs along with frequent courses of steroids [4]. Sometimes, patients may experience progressive warmth, swelling, tenderness, edema, and erythema mimicking deep vein thrombosis, cellulitis, and ruptured baker’s cyst. Thus, every possibilities should be meticulously searched and excluded [5], and any sterile and recurrent pyomyositis in the patient with underlying autoimmune inflamatory conditions with high disease activity should be suspected as aseptic pyomyositis and therefore should be treated aggressively for the primary disease.

## Conflict of interest

None.

## Funding

Not applicable.

## Ethical approval

Ethical approval was obtained from review board of national center for rheumatic diseases via letter number CR- 03/23.

## Consent

Informed written consent was obtained from the patient for use of the pictures and publish her case report.

## Guarantor

Dr Binit Vaidya is nominated as the guarantor for this report.
